# Lemierre Syndrome as a Complication of Laryngeal Carcinoma

**DOI:** 10.5811/cpcem.2017.12.36442

**Published:** 2018-01-24

**Authors:** Christopher Colbert, Molly McCormack, Wesley Eilbert, Lynea Bull

**Affiliations:** * University of Illinois at Chicago, College of Medicine, Department of Emergency Medicine, Chicago, Illinois; † St. Louis University School of Medicine, St. Louis, Missouri

## Abstract

Lemierre syndrome is a rare condition characterized by a septic thrombophlebitis of the internal jugular vein with septicemia and metastatic foci of infection. It typically occurs as the result of an infection in the head and neck, most commonly pharyngitis. For reasons that are unclear, the incidence of Lemierre syndrome has been increasing over the past 15 years. Diagnosis of Lemierre syndrome is often delayed, and identification of internal jugular vein thrombosis is often the first indicator of its presence. We report a case of Lemierre syndrome associated with a laryngeal carcinoma.

## INTRODUCTION

Lemierre syndrome is a rare condition characterized by a septic thrombophlebitis of the internal jugular (IJ) vein. This potentially fatal disorder results in bacteremia usually caused by *Fusobacterium necrophorum*, a gram-negative anaerobe that is a normal part of the oropharyngeal flora.^1^ Most cases of Lemierre syndrome begin as an oropharyngeal infection, though primary infections in the chest, teeth, sinuses, and ears may also serve as sources.^2^ Other rare causes include trauma and head and neck malignancies.^3^–^7^ We present a case of Lemierre syndrome associated with a laryngeal carcinoma.

## CASE REPORT

A 41-year-old man with a past medical history of squamous cell carcinoma of the glottis treated with radiation therapy five years earlier presented to our emergency department (ED) complaining of one week of progressive right-sided neck pain and swelling with fever up to 38.9^°^Celsius (C) (102.1^°^ Fahrenheit). His symptoms had been preceded by three months of throat pain, odynophagia, and right otalgia. A positron emission tomography performed at another institution one month before had revealed a lesion concerning for possible recurrence of the tumor. One week prior to presentation he had been prescribed a one-week course of cephalexin for a “neck infection” at another ED. He was afebrile (37.0^°^C) on examination with a 3 cm fluctuant neck mass palpable in his right anterior neck. The remainder of his examination was unremarkable.

**Image 1:**
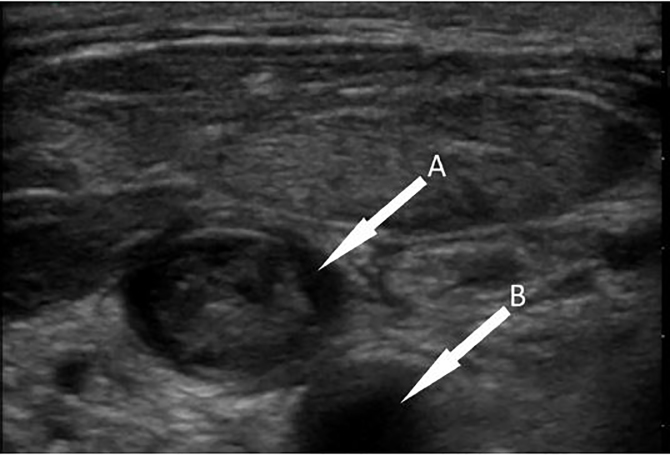
Point-of-care ultrasound of the right internal jugular vein (A) showing echogenic material in the lumen adjacent to the common carotid artery (B).

**Image 2:**
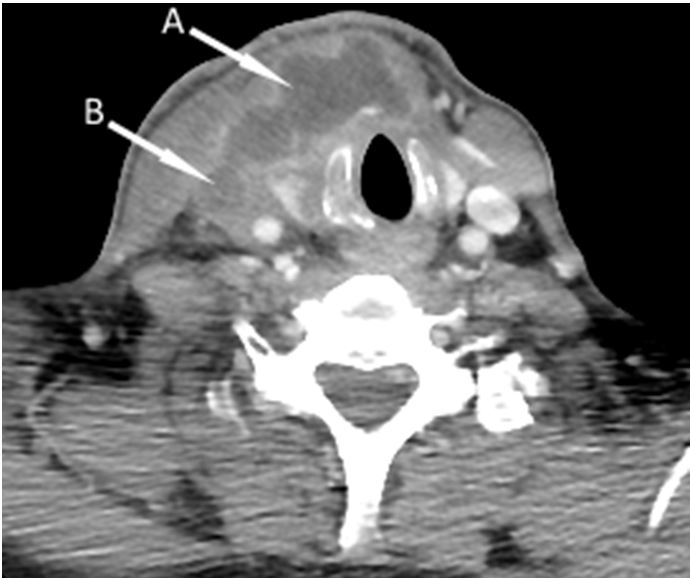
Contrast-enhanced computed tomography of the neck showing a soft tissue fluid collection (A) anterior and lateral to the trachea, and thrombus in the right internal jugular vein (B)

Laboratory analysis revealed a white blood cell count of 17,300/μl, and blood cultures were sent. A point-of- care ultrasound was performed, which showed echogenic material in a noncompressible right IJ vein (Image 1) – findings consistent with thrombophlebitis. A contrast-enhanced computed tomography (CT) of the neck later confirmed thrombosis of the right IJ vein extending to the subclavian vein, as well as a peripherally enhancing fluid collection in the right neck anterior and lateral to the trachea (Image 2). Needle aspiration of this fluid collection yielded purulent fluid that was sent for culture. Empiric antibiotic therapy was started with vancomycin and piperacillin/tazobactam, and the patient was started on enoxaparin for anticoagulation. A CT of the chest performed the next day revealed multiple bilateral, ground-glass opacities suspicious for infectious processes.

On hospital day three the patient underwent direct laryngoscopy, and biopsies of a glottic mass confirmed the diagnosis of squamous cell carcinoma. Blood cultures sent from the ED grew no organisms, though the fluid aspirated from the right neck fluid collection grew *Fusobacterium nucleatum* on hospital day six. At the recommendation of the infectious disease service, the patient’s antibiotic regimen was changed to ampicillin/sulbactam. The patient was discharged on hospital day 11 to complete a six-week course of antibiotic therapy with ertapenem and anticoagulation with enoxaparin. A contrast-enhanced CT of the neck performed 20 days after the initial scan showed no significant change in the right IJ vein thrombus. Ultimately the patient underwent total laryngectomy for treatment of his malignancy.

CPC-EM Capsule

What do we already know about this clinical entity?

Lemierre syndrome, a septic thrombophlebitis of the internal jugular vein, is a rare condition. There is frequently a delay in making its diagnosis.

What makes this presentation of disease reportable?

Lemierre syndrome typically occurs as a result of an infection in the head and neck. This is only the fourth reported case occurring in conjunction with a primary head and neck tumor.

What is the major learning point?

Lemierre syndrome may occur in the setting of a primary head and neck malignancy. Point-of-care ultrasound can be used in the emergency department to expedite its diagnosis.

How might this improve emergency medicine practice?

A timely diagnosis of Lemierre syndrome can be assisted by recognizing an active head and neck malignancy as a risk factor for its presence and the use of point-of-care ultrasound.

## DISCUSSION

Lemierre syndrome is classically characterized by the following: 1) primary infection in the oropharynx; 2) septicemia documented by at least one positive blood culture; 3) clinical or radiographic evidence of IJ vein thrombosis; and 4) at least one metastatic focus of infection (though several variations of this definition have been proposed).^8^,^9^ Lemierre syndrome is primarily a disease of young people, with the majority of cases occurring in the first three decades of life.^2^ With the introduction of antibiotics for the treatment of streptococcal pharyngitis in the 1940s, the incidence of Lemierre syndrome fell dramatically. Since the start of the 21^st^ century there has been a noted increase in the reporting of Lemierre syndrome.^10^–^12^ While unclear, the cause of this increase may be due to increased recognition of the disease, more stringent use of antibiotics for oropharyngeal infections, and increasing antibiotic resistance.^13^

*Fusobacterium necrophorum* is the responsible pathogen in over 80% of cases of Lemierre syndrome.^1^,^14^ Other reported pathogens include other *Fusobacterium* species, *Eikenella*, *Bacteroides*, *Streptococcus*, and *Staphylococcus* species.^13^ One review found blood cultures to be negative in 12.8% of cases, as with our case.^14^ The condition typically begins as a sore throat, followed days later by unilateral neck pain. Fever is the most common physical exam finding, present in 92% to 100% of cases, and approximately half of all patients will have a tender or swollen neck.^14^,^16^ The lungs are the most common site of metastatic infection, present in 80% to 97% of cases, followed by the joints.^8^,^14^

The diagnosis of Lemierre syndrome is often delayed, probably due in part to its relative rarity. One review reported an average delay of five days from the time of admission until diagnosis was made.^17^ Identification of thrombophlebitis of the IJ vein is the first hard evidence to suggest Lemierre syndrome in many patients.^17^ Contrast-enhanced CT is considered the imaging study of choice for this purpose by several authors.^8^, ^17^–^21^ As with our case, ultrasound offers a rapid, low-cost, noninvasive method of imaging the IJ vein. Ultrasound is limited by its ability to visualize recently formed, unorganized clot and incomplete evaluation of tissue adjacent to the mandible, clavicle, and skull base.^18^ As emergency physicians become more comfortable with the use of point-of-care ultrasound for various indications, its use in the future for rapid screening for Lemierre syndrome in the ED will likely increase.

Prolonged antibiotic therapy constitutes the mainstay of treatment of Lemierre syndrome. Since many *F. necrophorum* strains have beta-lactamase activity, most authorities recommend empiric treatment with clindamycin or a beta-lactamase resistant penicillin with good anaerobic coverage such as ticarcillin/clavulanate or ampicillin/sulbactam.^15^,^16^, ^21^,^22^ The use of anticoagulation is controversial and no controlled studies exist, though more recent retrospective studies have advocated for its use.^23^,^24^

The infection in our case most likely originated at the site of the malignancy where a loss of mucosal integrity allowed for entry of the pathogen. Thrombogenesis was further promoted by the presence of an active malignancy. To our knowledge, this is only the fourth reported case of Lemierre syndrome occurring in conjunction with a primary head and neck tumor.^5^–^7^

## CONCLUSION

While it remains rare, Lemierre syndrome seems to be occurring with increasing frequency in this century. There is frequently a delay in making its diagnosis. The presence of an active malignancy in the head and neck may be a risk factor for its occurrence. Recognition of patient risk factors and typical history and physical examination findings are crucial to expedite its identification. The use of point-of-care ultrasound provides emergency physicians with a rapid and noninvasive means to help identify this condition.
